# The Spanish Familial Pancreatic Cancer Registry (PANGENFAM): a decade follow-up of individuals at high-risk for pancreatic cancer

**DOI:** 10.1007/s10689-024-00388-x

**Published:** 2024-05-16

**Authors:** Julie Earl, Raquel Fuentes, María E. Castillo Sanchez, Ana García García de Paredes, María Muñoz, Alfonso Sanjuanbenito, Eduardo Lobo, Alejandra Caminoa, Mercedes Rodríguez, Emma Barreto, Jorge Villalón López, Ignacio Ruz-Caracuel, Sergio López Durán, José Ramón Foruny Olcina, Bárbara Luna Sánchez, Sonia Camaño Páez, Ana Torres, Javier Blázquez, Enrique Vázquez Sequeros, Alfredo Carrato

**Affiliations:** 1 Ramón y Cajal Health Research Institute (IRYCIS), Carretera Colmenar Km 9,100, 28034 Madrid, Spain; 2 The Biomedical Research Network in Cancer (CIBERONC), Av. Monforte de Lemos, 3-5. Pabellón 11. Planta 0, 28029 Madrid, Spain; 3 Biobank and Biomodels Platform, Spanish National Biobanks Network (ISCIII Biobank Register No. B.0000678), ISCIII Research and Development Platforms in Biomedicine and Health Sciences, BioBank Hospital Ramón y Cajal-IRYCIS, Ramón y Cajal Health Research Institute (IRYCIS), Carretera Colmenar Km 9, 100, PT20/004528034 Madrid, Spain; 4 Medical Oncology Department, Hospital Universitario Ramón y Cajal, IRYCIS, 28034 Madrid, Spain; 5 Gastroenterology and Hepatology Department, Hospital Universitario Ramón y Cajal, Madrid, Spain; 6 Centro de Investigación Biomédica en Red de Enfermedades Hepáticas y Digestivas (CIBERehd), Instituto de Salud Carlos III, Madrid, Spain; 7 Radiology Department, Hospital Universitario Ramón y Cajal, Madrid, Spain; 8 Pancreatic and Biliopancreatic Surgery Unit, Hospital Universitario Ramón y Cajal, Madrid, Spain; 9 University of Alcalá, Madrid, Spain; 10 Department of Pathology, Hospital Universitario Ramón y Cajal, 28034 Madrid, Spain; 11 Pancreatic Cancer Europe, Brussels, Belgium

**Keywords:** Pancreatic cancer, Biobanking, Early diagnosis, High-risk individuals, Screening

## Abstract

The Spanish Familial Pancreatic Cancer Registry (PANGENFAM) was established in 2009 and aims to characterize the genotype and phenotype of familial pancreatic cancer (FPC). Furthermore, an early detection screening program for pancreatic ductal adenocarcinoma (PDAC) is provided to healthy high-risk individuals from FPC and hereditary pancreatic cancer families (first-degree relatives). This article describes our experience over the last 10 years in high-risk screening. Hereditary and familial pancreatic cancer families were identified through the oncology and gastroenterology units. High-risk individuals underwent annual screening with endoscopic ultrasound (EUS) and magnetic resonance (MRI) from age 40 or 10 years younger than the youngest affected family member. Results: PANGENFAM has enrolled 290 individuals from 143 families, including 52 PDAC cases and 238 high-risk individuals. All high-risk individuals eligible for screening were offered to enter the surveillance program, with 143 currently participating. Pancreatic abnormalities were detected in 94 individuals (median age 53 years (29–83), with common findings including cystic lesions and inhomogeneous parenchyma. Imaging test concordance was 66%. Surgical intervention was performed in 4 high-risk individuals following highly suspicious lesions detected by imaging. PANGENFAM is a valuable resource for science innovation, such as biobanking, with clinical and imaging data available for analysis. For high-risk families, it may offer a potential for early diagnosis. Collaboration with other national and international registries is needed to increase our understanding of the disease biology and to standardize criteria for inclusion and follow-up, optimizing cost-effectiveness and efficacy.

**Supplementary Information:**

The online version contains supplementary material available at 10.1007/s10689-024-00388-x.

## Introduction

Pancreatic ductal adenocarcinoma (PDAC) represents the third leading cause of cancer-related mortality in the European Union and the United States [1, 2]. The impact of PDAC on public health is substantial, with projections indicating that it will ascend to the second position by 2030, surpassing breast, prostate, and colorectal cancers [3]. The grim reality of PDAC lies in its late-stage diagnosis, with approximately 7.2% of patients surviving five years following diagnosis [4] and its resistance to many types of therapy. Surgical resection remains the sole hope for cure or improved prognosis, yet it remains a viable option for only 15–20% of patients, and, dishearteningly, two-thirds will experience disease recurrence after surgery[5].

Thus, new approaches for early PDAC detection are desperately needed to improve the survival rate of patients. Population-wide screening for PDAC is not feasible due to its relatively low incidence. An estimated 4–10% of PDAC have a familial or hereditary background [6, 7]. Around 10–13% of these families carry germline mutations in DNA repair genes such as BRCA2, CDKN2A and mismatch repair genes related with Lynch syndrome. In the absence of a broadly defined high-risk population, early detection efforts currently concentrate on individuals at high risk of developing PDAC that could benefit from screening strategies that favor early detection during a potentially curable phase. In the context of familial pancreatic cancer (FPC), the risk of PDAC development escalates proportionally with the number of affected family members, with a standard hazard ratio of 32 with three affected family members, emphasizing the hereditary nature of the disease [8–11]. A family is considered to have FPC when at least one pair of first-degree relatives are afflicted with no known genetic basis.

This article presents a comprehensive review of the Spanish Familial Pancreatic Cancer Registry (PANGENFAM), established in 2009, with the dual mission of characterizing the phenotypic and genetic aspects of FPC, as well as providing a screening program for high-risk relatives [12]. The screening program consists of annual endoscopic ultrasound (EUS) and magnetic resonance imaging (MRI)., complemented with endoscopy ultrasound-guided biopsy when suspicious lesions are detected. High-risk individuals are managed by a multidisciplinary team including specialist clinicians involved in the management of pancreas pathologies.

## Methods

### Study design

This study is part of the ongoing Spanish Familial Pancreatic Cancer Registry, a prospective cohort study conducted in a university hospital in Spain (Hospital Universitario Ramon y Cajal). All participants gave written informed consent prior to enrolment. The study and all subsequent amendments to the study were approved by the local ethics committee.

## Participants

PDAC cases and high-risk individuals from FPC families with the following phenotype or characteristics are included in the registry: 1) FPC families with  ≥ 2 affected first or second degree relatives; 2) Hereditary breast and ovarian cancer (HBOC) families with at least one case of PDAC; 3) Families with ATM mutation and at least one case of PDAC; 4) Familial atypical multiple mole melanoma (FAMMM) families with at least one case of PDAC; 5) Hereditary Non Polyposis Colorectal Cancer (HNPCC) or Lynch Syndrome families with at least one case of PDAC; 6) Peutz Jeghers families; 7) Hereditary Pancreatitis (with pathogenic variants in the genes PRSS1 and SPINK1); and 8) Families with PDAC cases diagnosed at  ≤ 50 years of age.

## Surveillance protocol

High-risk individuals are offered to enter a screening program consisting of the construction of a family tree with at least 3 generations and an updated clinical record, with annual EUS, MRI, and blood sample collection from age 40 or 10 years younger than the youngest PDAC affected family member. The timing of the imaging tests may be shortened in case of findings that justify closer follow-up after evaluation of the case by a multidisciplinary committee composed of gastroenterologist, oncologists, radiologists, pathologists, and surgeons. When suspicious lesions are detected EUS, guided tissue acquisition is performed. CT was used as a screening modality in high-risk individuals from 2011 until the beginning of 2013, since then, only MRI and EUS are used routinely and CT is requested only in specific cases. A screening round was defined as imaging tests, either EUS, MRI or CT, performed within 6 months of one another. Normal imaging was defined as consistently normal pancreatic imaging during screening. The detection of any pancreatic abnormality (cysts, inhomogeneous pancreas parenchyma etc.) was defined as a pancreatic abnormality, independently of the imaging modality.

## Data storage and confidentiality

Data is stored in a pre-designed REDCap database. Data collected for all individuals include demographics, clinical information and epidemiological data (tobacco smoking, pancreatitis, diabetes, overweight, cancer family history); for PDAC cases clinical, histological, imaging, TNM staging, blood analysis results, first-line treatment prescribed and follow-up data (stage, ECOG, treatment, imaging and blood analysis); and for high-risk individuals, clinical information, imaging and blood analysis results.

## Statistical analysis

The Chi-square test was used to evaluate differences in non-continuous variables (gender) between different groups and t-test for differences in continuous variables (age). Statistical analysis was performed with the RStudio program [19].

## Results

### Familial and hereditary pancreatic cancer families included in PANGENFAM registry

Since 2009, 290 individuals from 144 families have been enrolled in PANGENFAM, including 52 PDAC cases and 238 high-risk individuals. The family phenotypes are shown in Table 1. The majority of families (58%) are classified as FPC with at least 2 first degree affected relatives.

**Table 1:** Distribution of family phenotypes included in the PANGENFAM registry

Family phenotype	Number of families
FPC	84
HBOC	32
PJS	3
HP	2
FAMMM	2
PDAC ≤ 50 years	9
Lynch syndrome	5
HNPCC	5
ATM	2
TOTAL	144

*FPC* families with  ≥ 2 affected first degree relatives; *HBOC* hereditary breast and ovarian cancer families with at least one case of PDAC; *Lynch Syndrome* pathogenic germline mutation in mismatch repair genes MLH1, MSH2, MSH6 and PMS2 or EpCAM with at least one case of PDAC; *HNPCC* Hereditary Non Polyposis Colorectal Cancer families (complies with Amsterdam I or II clinical criteria) with at least one case of PDAC; *PDAC  ≤ 50 years* families with PDAC cases diagnosed at  ≤ 50 years of age; *PJS* Peutz Jeghers families; *FAMMM* Familial atypical multiple mole melanoma (FAMMM) families with at least one case of PDAC; *ATM* Families with ATM mutation and at least one case of PDAC; *HP* Hereditary Pancreatitis

### Pancreatic anomalies identified during screening

Of the 238 high-risk individuals, 189 (79%) were eligible and consented to enroll in the screening program. The median age at the start of screening was 50 years (29–83) and 62% were females. Most high-risk individuals in screening were from FPC families (58%) and HBOC families (21%). On baseline imaging when entering the screening program, of the 189 individuals, 141 (78%) had a normal pancreas and 39 (22%) had pancreatic findings. These pancreatic findings included: 14 cysts (4 suspected intraductal papillary mucinous neoplasms-IPMN), 8 inhomogeneous pancreatic parenchyma, 1 solid lesion, 1 dilation of the main pancreatic duct, 1 chronic pancreatitis, 1 pancreatic steatosis, 1 pancreas divisum. There were no significant differences regarding age and gender distribution between individuals with normal and abnormal baseline imaging (51 years (29–83, 60% females) normal imaging vs. 53 years (29–77, 72% female) abnormal pancreas imaging).

Of the 189 individuals that initiated screening, 68% underwent at least a second imaging round. In total, 627 rounds of screening were performed in these individuals, ranging from 1 round only to 12 rounds of screening. Excluding individuals that had only undergone one round of follow-up, the median time in follow-up was 3.81 years (0.99–11.33). Currently, there are 143 high-risk individuals in active follow-up.

**Fig. 1:**
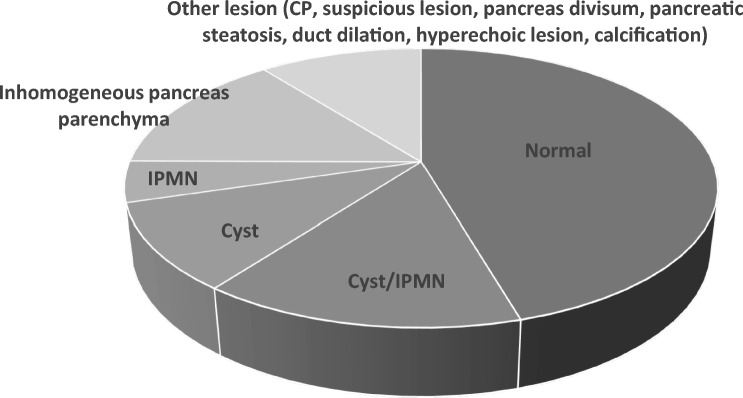
Consensus diagnosis of pancreas imaging of the 180 individuals in follow-up

During follow-up imaging, a normal looking pancreas was consistently identified in 86 (48%) individuals and some type of pancreatic finding was detected in 94 (52%) individuals. Pancreatic cysts were identified in 57 high-risk individuals (32%). Of these, the lesion was consistently identified as a cyst during follow-up in 20 individuals (11%). IPMN were identified on entry into screening and consistently throughout screening in 9 individuals (5%). Inhomogeneous pancreatic parenchyma was detected in 28 individuals and chronic pancreatitis imaging stigmas were detected 4 individuals. Pancreatic anomalies found by imaging are summarized in Fig. 1.

**Fig. 2:**
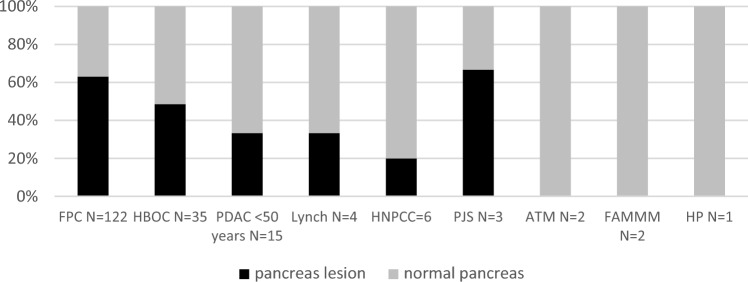
Frequency of normal and abnormal pancreas imaging during screening according to family phenotype

Imaging findings according to family phenotype are summarized in Fig. 2, 63% of high-risk individuals from FPC families and 49% from HBOC families had abnormal pancreas imaging during screening. The 9 lesions consistently identified as IPMN were only found in FPC families, whereas, the 19 lesions consistently identified as cysts were mainly found in FPC families (58%) and HBOC families (32%), as well as PJS (5%) and Lynch syndrome (5%). Of the 15 high-risk individuals from PDAC < 50 years families, 66% had abnormal pancreas imaging, the pancreatic abnormalities included 1 individual with a cyst, another with a cyst/IPMN, and 2 other individuals with an inhomogeneous pancreas. The fifth individual had a highly suspicious image on screening entry and finally underwent a surgical resection (Table 2). One individual with Lynch syndrome had a cyst and another individual with HNPCC had an inhomogeneous pancreas. The 2 high-risk individuals from FAMMM, 2 from ATM and one from a HP family had normal pancreas imaging. Of the 3 high-risk from PJS families, one had a cyst and the other 2 had a normal pancreas.

**Table 2:** Summary of suspicious lesions detected during the screening program that underwent a surgical resection. *previously reported [14, 15]

High-risk individual	Date (intervention)	Intervention results	Final diagnosis
FPC Family: female with 1 first degree and 1 s degree relative with PDAC, entered high-risk screening age 47*	2012 (round 1 screening)	CT: 7 mm hypodense focal lesion at the body-tail junction EUS: cystic lesion of 8.4 × 7.9 mm in pancreatic body with a mucinous tumor appearance MRI: 1 cm diameter cystic lesion	cyst/IPMN
	2013 (round 2 screening)	MRI: cystic lesion of 1 cm in diameter unchanged from previous imaging	cyst/IPMN
	2014 (round 3 screening)	MRI: pancreatic cyst, unchanged from previous study	cyst/IPMN
	2014 (biopsy)	EUS + FNA	Suspicious of carcinoma
	June 2014 (surgery)	Corporocaudal pancreatotomy and splenectomy (age 49)	Multiple PanIN-1 foci
	2015–2022 (post-surgery follow-up)	Annual CT and/or EUS and/or MRI. Last April 2023, aged 58	Changes consistent with a surgical intervention
FPC Family: Female with 1 first degree and 2 s degree relatives with PDAC, entered high-risk screening aged 39*	2015 (round 1 screening)	CT: No focal lesion in the pancreas EUS: An ill-defined solid tumor of 5mmx4mm located at the tail-body junction MRI: Normal	Suspicious solid lesion
	2015 (biopsy)	EUS + FNA	Epithelial lesion with mild atypia, suspicious of malignancy
	2015 (surgical intervention)	Corporocaudal pancreatotomy and splenectomy, age 40	Multifocal PanIN 1a and 1b lesions
	2016–2023 (post-surgery follow-up)	Annual CT and/or EUS and/or MRI. Last follow-up February 2023, age 48	Changes consistent with a surgical intervention
FPC Family: female with 2 s degree relatives with PDAC, entered high-risk screening aged 47*	2012 (round 1 screening)	CT: Normal EUS: Small cystic lesion MRI Normal	Cyst
	2013 (round 2 screening)	EUS: Tumor in tail of pancreas	
	2013 (biopsy)	EUS + FNA	Suspicious solid lesion, compatible with a neuroendocrine tumor
	2013 (surgical intervention)	Distal pancreatectomy, age 48	Grade 2 neuroendocrine tumor
	2014–2022 (post-surgery follow-up)	Annual CT and/or EUS and/or MRI. Last follow-up December 2022, aged 57	Changes consistent with a surgical intervention
PDAC < 50 years Family: male with 1 first degree relative with PDAC diagnosed aged 48 years and 1st degree relative diagnosed with liver metastasis of a non-identified primary tumor, entered high-risk screening aged 54	Oct 2020 (round 1 screening)	CT: Fatty infiltration of the pancreatic gland and two nodules EUS: Poorly delimited hypoechogenic area of 18–19 mm in the uncinate process. Dilation of the pancreatic duct MRI: Dilatation of the main pancreatic duct at the level of the uncinate process-head of the pancreas, with a hypodense area in the uncinate process	Solid lesion
	Diagnostic imaging	CT: nodular appearance of the pancreatic parenchyma. Retrograde dilatation of the main pancreatic duct reaching about 13 mm	
	Jan 2021 (biopsy)	EUS + FNA	Solid lesion
	Feb 2021 (surgical intervention)	Surgery: 11/02/2021, cephalic duodenopancreatectomy, age 54. Adjuvant treatment with modified FOLFIRINOX	Ductal adenocarcinoma, G2, pT2N2(5/22 lymph nodes)
	2021–2023 (post-surgery follow-up)	Annual CT and/or EUS and/or MRI. LAST follow-up August 2023, age 56	Changes consistent with a surgical intervention, NED

Eight high-risk individuals had more than one pancreatic finding, and each finding was counted separately. In 6 cases, the high-risk individual was first diagnosed with an inhomogeneous pancreas and was subsequently diagnosed with a cyst. One individual was diagnosed with chronic pancreatitis in round 3 of imaging at age 50 and then with a cyst 6 years later. Another individual was first diagnosed with pancreatic duct dilation aged 64 and was finally diagnosed with an IPMN aged 66. There were no significant differences regarding age and gender distribution between those individuals with normal and abnormal imaging on follow-up. The mean age at diagnosis of inhomogeneous pancreas parenchyma was significantly lower than for cysts and IPMN; (48 years (29–67) vs. 55 years (38–83) and 55.5 years (30–83), respectively.

The overall concordance rate between imaging tests was 66% and the best concordance rate was seen with normal pancreas imaging. Within the non-concordant imaging rounds, the majority (85 rounds, 14%) were diagnosed as a non-normal looking pancreas vs. a normal looking pancreas, these abnormalities were mainly inhomogeneous pancreas parenchyma versus a normal looking pancreas. There was a discrepancy between the pancreatic lesion being a cyst or an IPMN in 28 rounds (4%), a normal looking pancreas and a cyst in 6 rounds (1%) and a normal looking pancreas and IPMN and 10 rounds (1.6%).

### Surgical intervention due to a suspicious lesion

After discussion by the multidisciplinary team, 4 high-risk individuals have undergone a surgical resection due to the detection of highly suspicious pancreatic lesions. These lesions were detected on baseline imaging in 2 individuals, which were eventually resected with the identification of Pan-IN-1 lesions in the resected specimen. Another individual underwent a surgical resection of a pancreatic neuroendocrine tumour detected during follow-up. These 3 cases have previously been reported and they are still under follow-up and are currently disease free [14, 15]. A solid adenocarcinoma was detected in another individual, which was surgically resected, and the patient underwent adjuvant treatment with modified FOLFIRINOX, and currently has no evidence of disease. The imaging findings that led to the surgical intervention are summarized in Table 2. All individuals were negative for targeted panel sequencing using a custom designed panel of 66 familial cancer related genes [10]

### Extrapancreatic findings

No extra-pancreatic lesions were identified in 33% of high-risk individuals and a total of 236 extra-pancreatic lesions were detected in the remaining 67% of individuals. The most frequent extra-pancreatic lesions were hepatic cysts (23) and renal cysts (21), followed by biliary cysts (8), cholelithiasis (6), hepatic steatosis (4), vesicular polyp (5), focal hepatic lesions (3), accessory spleen (3) and hiatal hernia (2%). A full list of extrapancreatc lesions identified is available in Supplementary Table 1. One duodenal ampuloma was identified by EUS in an 83-year-old female. It was successfully resected endoscopically and definitively histology revealed a low-grade tubular adenoma. The same patient was subsequently diagnosed with a papillary urothelial carcinoma (pTaG3) a few months later.

### Updated PANGENFAM inclusion and screening criteria

Based on our 10-year experience of the follow-up of high-risk individuals and according to the recent international guidelines [16, 17], the inclusion criteria for high-risk screening have been updated as follows: (1).  ≥ 2 relatives with pancreatic cancer on the same side of the family where 2 affected individuals are first degree relatives and at least 1 affected individual is a first degree relative of the high-risk individual considered for screening. (2). Peutz-Jeghers syndrome (carriers of germline pathogenic mutation in STK11/LKB1), FAMMM (carriers of pathogenic germline CDKN2A mutation), Hereditary Pancreatitis (carriers of a pathogenic mutation in PRSS1), and carriers of a germline pathogenic mutation in BRCA1, BRCA2, with at least one first degree relative with pancreatic cancer. (3). Carriers of germline pathogenic mutation in PALB2, ATM, MLH1, MSH2, MSH6, PMS2 or EPCAM with a first degree relative with pancreatic cancer.

Regarding the screening protocol, this is will be in accordance with the recommendations of the CAPS consortium, starting at age 50 or 10 years before the diagnosis of the youngest relative with PDAC in the family. With the following specific exceptions: individuals with Peutz-Jeghers syndrome (STK11 mutation) from 35 years of age, FAMMM (CDKN2A mutation) from 40 years of age and Hereditary pancreatitis (PRSS1 mutation) from 40 years of age [18]. Basal imaging using EUS and MRI will be performed in all high-risk individuals that enter screening, due to their complementary nature in high-risk screening. From then on, imaging will alternate between EUS or MRI according to the CAPS recommendation or participant's preferences [16–18]. Regarding interpretation of the imaging tests, the standard reports developed by the PRECEDE consortium for EUS [19] and MRI [20] will be used.

### Description of the costs of screening

189 high-risk individuals had some imaging performed within the screening program. 1160 imaging tests were performed since the study beginning in in 2010, including 522 MRI, 528 EUS, 70 CT and 40 other types of imaging, mainly thoracic CT and abdominal echography. Of all imaging tests, 66% were considered as a normal looking pancreas and 44% an abnormal pancreas. The total cost of screening these individuals via imaging was 546,000€, based on approximate costs of EUS, MRI and CT imaging in the public health system in Spain. In accordance with the current screening guidelines and based on our own experience, the approximate cost of only screening individuals more than 50 years of age is 328,000€, which equates to a saving of 40%. Whereas, the cost of only screening individuals greater than 50 years of age and alternating between EUS and MRI annually, is 177,900€, a saving of 67% based on our current screening criteria and protocol. The approximate annual cost of screening the 143 individuals in active screening in our hospital when applying the new criteria is 71,500€.

## Discussion

The benefits of pancreas surveillance in carriers of pathogenic germline variants is clearly established [15, 21–25]. A recent study from the Dutch Pancreatic Cancer Group showed that PDAC surveillance in germline carriers of CDKN2A/p16 pathogenic variant resulted in early detection with resectable disease and better overall compared survival compared with cases diagnosed outside of a surveillance program [26]. In fact, a shift towards more genetic testing of PDAC cases with a family history has occurred over the last 10 years, mainly due to the reduced cost and ease of genetic testing using panels, and the fact that pathogenic variants have been found in patients with no obvious familial cancer syndrome [27]. The current clinical guidelines for germline testing in FPC families in our centre are the following:  ≥ 3 cases with pancreatic adenocarcinoma in the same side of the family and  ≥ 2 first-degree relatives with pancreatic cancer. The minimum set of genes tested include BRCA1, BRCA2, MLH1, MSH2, MSH6, ATM, PALB2, CDKN2A, CHEK2, TP53 and STK11.

High-risk individuals from FPC families are also recommended to participate in screening programs to detect pancreatic cancer or high-risk precursor lesions at a potentially resectable stage. However, the need for follow-up in FPC individuals is controversial due to the low diagnostic yield [22, 28]. Although, pancreatic lesions identified in high-risk individuals tend to show a rapid progression, with advanced PDAC detected during surveillance [29, 30]. In our cohort, the most commonly detected lesions were cysts or IPMN found in 30% of individuals. Screening has successfully detected and treated solid lesions in 4 high-risk individuals, including 3 resectable exocrine lesions, 1 PDAC and 2 PanIn, and 1 neuroendocrine tumor. Although not all these resected lesions comply with CAPS pathological screening targets of stage I PDAC and high-grade precursors lesions such as PanIN or IPMN [16]. High-risk lesions that require clinical intervention are rarely found when screening these individuals and surgical intervention was performed in 2.6% of individuals from our registry, which is in line with other reported studies [29, 31, 32]. Furthermore, a recent meta-analysis showed that the risk of “low-yield” surgery during PDAC surveillance, defined as no high-grade precursor lesions or T1N0M0 tumors was low, thus advocating for surveillance in these individuals [33]. Although, late stage PDAC detection during surveillance, T2–4 with or without metastasis, remains a frequent occurrence [30]. EUS and MRI are complementarily imaging modalities used in the majority of screening, with a similar diagnostic yield [28].

Compared with other international registries that offer screening, the criteria applied in our hospital were very broad and adopted a more liberal approach, in terms of family phenotype, age at start screening and screening intensity. In line with our experience in high-risk screening and the recent literature, our screening protocol has been updated to a more conservative and targeted approach. This includes increasing the age at start screening in FPC families to age 50 [14, 28] and alternating between MRI and EUS in high-risk individuals from FPC families. In the context of our screening protocol, this translates to a cost saving of around 67%, with a total annual cost of high-risk screening of less than 100,00€. This is important, as if a PDAC high-risk screening program is to be offered in the public health system, the health benefit should outweigh the risk and also be cost-effective. There are no obvious gender disparities among PDAC cases, with around 48% of patients being female and 52% male [34]. However, there seems to be a disparity amongst those who take up high-risk surveillance, within our PANGENFAM registry (62% females) as well as globally. A recent study showed that the majority of participants in the high-risk screening consortium were female (65.9%), and also reported disparities in race and ethnicity [35], which needs to be addressed in the future in order that the population under surveillance accurately reflects the demographics of diagnosed cases.

Sensitive and specific biomarkers for early detection in high-risk groups are an important unmet need to improve the efficacy of screening programs to accurately diagnose high-risk precursor lesions or PDAC at a potentially curable stage. CA19-9 analysis was finally excluded from the follow-up program protocol due to the difficulty to interpret this analysis and effectively inform the high-risk of its significance in the context of high-risk screening. However, the clinical application of the liquid biopsy is likely to be an integral part of cancer diagnostics and management in the near future, with several potential biomarkers for early detection reported in the scientific literature [36–39]. Imaging biomarkers are transforming the way radiology contributes to cancer diagnosis by the extraction of quantitative and qualitative features from MRI and CT images that are undetectable to the naked eye [40]. Applications such as radiogenomics, the correlation of genomics and radiological studies, will ultimately improve patient and high-risk management [41]. Epigenomics is a relevant complimentary area that we are considering. We have serially collected clinical data, imaging, and blood test for biobanking with data on  smoking and drinking habits, BMI, pancreatitis and diabetes in high-risk individuals. The long term aim of our registry is to generate high-quality data from PDAC cases and high-risk individuals that can be used for Big Data science projects, by sharing anonymized data with international registries working on common aims [42].

## Conclusions

Screening high-risk individuals is recommended and can detect pancreatic lesions during a curable stage, although with a low diagnostic yield. Optimization of the screening protocol, particularly in high-risk individuals from FPC families, can make the program more cost effective, whilst still providing the maximum health benefit.

### Supplementary Information

Below is the link to the electronic supplementary material.

Supplementary file1 (DOCX 13 KB)
