# Supplementary material for: The Spanish Familial Pancreatic Cancer Registry (PANGENFAM): a decade follow-up of individuals at high-risk for pancreatic cancer
Source: Fam Cancer. 2024 May 16;23(3):383–92. doi: 10.1007/s10689-024-00388-x (PMC11254983; doi:10.1007/s10689-024-00388-x)
Supplement: Supplementary file 1 — Supplementary file1 (DOCX 13 KB) [file 10689_2024_388_MOESM1_ESM.docx]

**Supplementary Table 1.**

| **Extra pancreatic lesions** | **Number of lesions** | **% lesions** |
| --- | --- | --- |
| Normal | 60 |  |
| Renal cysts | 48 | 21 |
| Hepatic cysts | 54 | 23 |
| Biliary cysts | 18 | 8 |
| Spleen cysts | 4 | 2 |
| Adrenal cyst | 2 | 1 |
| Focal hepatic lesions | 8 | 3 |
| Hepatic steatosis | 10 | 4 |
| Cholelithiasis | 14 | 6 |
| Hepatic hemangiomas | 2 | 1 |
| Vertebral hemangioma | 3 | 1 |
| Accessory spleen | 6 | 3 |
| Enlarged lymph nodes | 4 | 2 |
| Jejunojejunal intussusception | 1 | 0 |
| Vesicular polyp | 11 | 5 |
| Renal angiomyolipoma | 2 | 1 |
| Duodenal ampuloma | 1 | 0 |
| Biliary sludge | 1 | 0 |
| Hiatal hernia | 5 | 2 |
| Intraabdominal collection | 1 | 0 |
| Adrenal nodule | 2 | 1 |
| Liver injury | 2 | 1 |
| Cholecystectomy | 5 | 2 |
| Spleen granuloma | 1 | 0 |
| Renal lithiasis | 2 | 1 |
| Biliary hamartoma | 1 | 0 |
| Intestinal diverticula | 2 | 1 |
| Free liquid in the peritoneum | 1 | 0 |
| Sinus cysts | 1 | 0 |
| Hepatic nodule | 2 | 1 |
| Hemothorax | 1 | 0 |
| Adrenal adenoma | 2 | 1 |
| Renal atrophy | 1 | 0 |
| Colon diverticulosis | 1 | 0 |
| Atheromatosis | 1 | 0 |
| Splenic hemangioma | 1 | 0 |
| Scoliosis | 2 | 1 |
| Follicular cysts | 1 | 0 |
| Arteriosclerosis | 1 | 0 |
| Liver disease | 1 | 0 |
| Solid liver nodules | 2 | 1 |
| Adrenal myelolipoma | 1 | 0 |
| Dilation of the biliary tract | 2 | 1 |
| Renal pelvicalyceal dilatation | 1 | 0 |
| Splenomegaly | 1 | 0 |
| Chronic gastritis | 1 | 0 |
| **Total** | **234** |  |
